# Risk factors and predictors of recurrence of febrile seizures in children in Nantong, China: a retrospective cohort study

**DOI:** 10.1186/s12887-024-04895-9

**Published:** 2024-07-01

**Authors:** Feifei Shen, Leijuan Lu, Youjia Wu, Guihai Suo, Yuqin Zheng, Xiuli Zhong, Xu Wang, Haiying Li

**Affiliations:** 1 Department of Pediatrics, Affiliated Hospital of Nantong University, No.20 Xisi Rd, Nantong, Jiangsu 226001 China; 2 Department of Pediatrics, Affiliated Haimen Hospital of Xinglin College, Nantong University, Nantong, Jiangsu Province China; 3 Department of Gynaecology and Obstetrics, Affiliated Hospital of Nantong University, Nantong, Jiangsu Province China; 4 Department of Science and Technology, Children’s Hospital of Nanjing Medical University, Nanjing, 210008 China

**Keywords:** Febrile seizures, Recurrence, Risk factors, Predictors

## Abstract

**Background:**

Although most children with febrile seizures (FS) have a favorable prognosis, some experience recurrence within 1–3 years. Age, peak temperature, and family history are now recognized as important risk factors for FS recurrence, yet studies in this area are lacking in China. This study aimed to investigate the risk factors for FS recurrence in children in Nantong, China, and to develop a prediction model.

**Methods:**

This retrospective cohort study analyzed 463 children diagnosed with febrile seizures (FS) who presented to the Affiliated Hospital of Nantong University between January 2015 and June 2020. Basic information, disease characteristics, and laboratory and imaging data were collected. A follow-up survey was conducted one year post-discharge to assess the recurrence status of FS in children. Univariate logistic regression and random forest models were used to identify and rank the predictive ability of risk factors for recurrence.

**Results:**

Of the 463 children with FS, 70 experienced recurrences within 1 year of discharge, resulting in a one-year recurrence rate of 15%. Age (OR = 0.61, 95% CI: 0.46, 0.80, *P* < 0.001), duration of the first episode (OR = 1.03, 95% CI: 1.00, 1.06, *P* = 0.040), and peak temperature (OR = 0.68, 95% CI: 0.47, 0.98, *P* = 0.036) were identified as independent risk factors for FS recurrence. Age had the highest relative importance in predicting FS recurrence, followed by the duration of the first episode, with an area under the ROC curve of 0.717.

**Conclusion:**

Young age and duration of the first seizure are important independent risk factors for FS recurrence and are key considerations for predicting recurrence. Further research is needed to confirm the potential use of Neutrophil–lymphocyte ratio (NLR) as a predictor of FS recurrence.

## Introduction

The American Academy of Pediatrics (AAP) defines febrile seizures (FS) as those occurring in children aged 6 months to 5 years with fever (≥ 100.4°F or 38℃) and without central nervous system infection [1]. The prevalence of FS is approximately 2%-5% in Western countries and slightly higher in Asian countries: 3%-5% in China, 5%-10% in India, and 6%-9% in Japan [2, 3]. While the majority of affected children have a good prognosis, a subset experience recurrences within 1–2 years [4. The World Health Organization (WHO) reported a 27.1% recurrence rate of FS in children in 1980, and subsequent studies have observed similar rates of around 30%, with some indicating a 15%-70% risk of recurrence in the first two years after the initial FS [5–7]. FS recurrence may delay language development and seems to be associated with the risk of epilepsy and mental disorders [8–10]. Moreover, studies have shown that children with underlying neurological abnormalities have a significantly higher risk of death within two years after their first FS [11]. The recurrence of FS not only impacts the growth and development of the child but also places a substantial financial and psychological burden on their families. For most parents, FS is a frightening event and one of the most common reasons for trips to the emergency room.

Age, peak temperature, and family history are now considered important risk factors for FS [7, 12–14]. The inflammatory pathways associated with fever may serve as a key trigger for FS in children. Tumor necrosis factor-alpha (TNFα), interleukin-6 (IL-6), and interleukin-1β (IL-1β) are commonly used inflammation indicators in assessing children with FS [15]. In recent years, neutrophil counts and neutrophil-to-lymphocyte ratios have been recommended because of their simplicity and cost-effectiveness [16, 17]. Several studies have investigated the association between the risk factors influencing the initial onset of FS and the recurrence of FS. Existing studies have validated the association of some of these risk factors with the recurrence of FS [5, 14, 18, 19]. However, the actual influence of some risk factors on the recurrence of FS remains controversial. For instance, a study by Kazemi et al. found that male children were more likely to recurrent, whereas a study by Nezami et al. showed no difference between males and females [6, 20].

FS recurrence imposes multiple physical, psychological, and economic burdens on children and parents. Currently, clinicians lack an objective assessment system to predict recurrence in children with FS, which affects the treatment and prognosis of these children. The incidence and risk factors of febrile seizures vary between ethnic groups [13, 21], and the risk factors and recurrence rates of febrile seizure recurrence may also exhibit regional and ethnic differences. Studies indicate that the recurrence rate of febrile seizures in southern Chinese children is lower compared to Western world [14]. There is limited research on recurrent febrile seizures in children from other regions of China. The aim of this study was to investigate the risk factors associated with FS recurrence in children in Nantong, China, and to further construct a prediction model using random forest model based on objective data, to screen out the most predictive risk factors, to identify children with a high likelihood of recurrence at an early stage and to improve the prognosis of these children.

## Subjects and methods

### Study subjects

The research screened 463 children with FS from January 2015 to June 2020 among children presenting with fever and convulsions at the Affiliated Hospital of Nantong University.

Inclusion criteria:

1. Admitted within 72 h of the onset.
2. Age 6 months to 6 years.
3. Diagnosed with FS for the first time.

Exclusion criteria:

1. Other causes of convulsions could not be ruled out, such as inherited metabolic disorders, developmental abnormalities of organ systems, etc.
2. Severe electrolyte disorders.
3. Missing data and lost visits.
4. History of epilepsy.
5. Complex FS.

### Data collection

From the medical records, we gathered essential details about children (including age, sex, birth weight, delivery mode, Feeding method, and family history of FS or epilepsy), disease characteristics (including duration of the first episode, highest temperature before the first episode, and number of seizures episodes prior to the visit), and clinical information (complete blood count, biochemical test, cranial computed tomography (CT), and electroencephalogram (EEG)) from the medical records. All blood tests were conducted on the consultation day.

FS recurrence was monitored through follow-up one year after children were discharge from the hospital. An experienced pediatrician obtained a comprehensive description of the seizure from the parents or witnesses. The recurrence of FS was determined based on the seizure characteristics and the presence of fever. Cases where an exact body temperature was not obtained through a thermometer are considered to have normal body temperature..

The study was approved by the Ethics Committee of the Affiliated Hospital of Nantong University (Approval No. 2022-K015-01).

### Statistical methods

Continuous variables were expressed as mean ± standard deviation and categorical variables as frequency (percentage). Continuous variables were compared between two groups using independent samples t-test and categorical variables were compared between two groups using chi-square test. Risk factors for recurrence of FS analyze using a univariate logistic regression model. Random forest predictive models were built using the "randomForest" package (R). Statistical analyses were performed using R software (version 4.1.3, R Development Core Team, Vienna, Austria), with the significance level set at a two-sided *P*-value < 0.05.

## Results

A total of 463 children with FS were included in this study, with 70 (15%) experiencing recurrence within 1 year of discharge while 393 did not experience recurrence. The mean age was 1.55 ± 1.10 years in the recurrent group, compare to 2.26 ± 1.58 years in the non-recurrent group, with a significant difference between the two groups (*P* < 0.001), as shown in Table 1. The mean duration of the first episode was 6.10 ± 6.90 min in the recurrent group, compare to 4.22 ± 6.51 min in the non-recurrent group, with a significant difference between the two groups (*P* = 0.037). Neutrophils count (6.34 ± 4.4410^9^/L) and NLR (3.56 ± 2.74) were significantly lower in the recurrent group than in the non-recurrent group (8.01 ± 5.7210^9^/L, 4.76 ± 5.12). In addition, peak temperature, neutrophil count, NLR, serum sodium and potassium were also significantly different between the two groups. The results are detailed in Table 2.

**Table 1:** Comparison of Baseline Data Between Recurrence and Non-recurrence Groups

Variable	Recurrence (*n* = 70)	Non-recurrence (*n* = 393)	t/χ^2^	P*
Age (years)	1.55 ± 1.10	2.26 ± 1.58	-4.629	** < *0.001***
Gender			0.360	0.549
Boys (n, %)	234 (59.5)	39 (55.7)		
Girls (n, %)	159 (40.5)	31 (44.3)		
Family history			0.360	0.549
Yes (n, %)	7 (10.0)	23 (5.9)		
No (n, %)	63 (90.0)	370 (94.1)		
Delivery mode			1.418	0.234
Vaginal (n, %)	32 (45.7)	150 (38.2)		
Cesarean (n, %)	38 (54.3)	243 (61.8)		
Feeding history			0.547	0.761
Breastfeeding (n, %)	39 (55.7)	201 (51.1)		
Formula feeding (n, %)	10 (14.3)	66 (16.8)		
Mixed feeding (n, %)	21 (30.0)	126 (32.1)		
Birth weight (kg)	3.43 ± 0.44	3.40 ± 0.48	0.482	0.630

^*^P-values in bold and italic indicate statistically significant differences

**Table 2:** Comparison of Disease Characteristics, Laboratory, and Imaging Data Between Recurrence and Non-recurrence Groups

Variable	Recurrence (n = 70)	Non-recurrence (n = 393)	t/χ^2^	*P*
Duration of first episode (min)	6.10 ± 6.90	4.22 ± 6.51	2.121	***0.037***
Peak temperature (℃)	39.10 ± 0.71	39.30 ± 0.72	-2.107	***0.036***
White blood cell count (10^9/L)	10.44 ± 9.14	11.33 ± 6.25	-1.007	0.314
Neutrophil count (10^9/L)	6.34 ± 4.44	8.01 ± 5.72	-2.763	***0.007***
Lymphocyte count (10^9/L)	2.40 ± 1.51	2.46 ± 1.56	-0.296	0.767
Red blood cell count (10^12/L)	4.57 ± 0.34	4.56 ± 0.43	0.045	0.964
Hemoglobin (g/l)	121.37 ± 10.19	122.69 ± 10.25	-0.992	0.322
Neutrophil–lymphocyte ratio	3.56 ± 2.74	4.76 ± 5.12	-2.871	***0.005***
Platelet count (10^9/L)	252.73 ± 86.43	256.16 ± 86.36	-0.309	0.758
CRP (mg/L)	12.35 ± 18.07	15.40 ± 23.70	-1.025	0.306
AST (IU/L)	46.69 ± 10.21	52.37 ± 72.34	-0.656	0.512
ALT (IU/L)	27.97 ± 9.60	31.34 ± 32.27	-1.690	0.092
LDH (IU/L)	607.34 ± 247.49	619.97 ± 288.75	-0.344	0.731
Serum potassium (mmol/L)	4.49 ± 0.52	4.36 ± 0.49	1.978	***0.048***
Serum sodium (mmol/L)	137.13 ± 3.79	135.85 ± 4.23	2.365	***0.018***
Serum magnesium (mmol/L)	0.90 ± 0.09	0.88 ± 0.12	1.550	0.122
Serum calcium (mmol/L)	2.41 ± 0.14	2.39 ± 0.14	1.098	0.273
Random blood glucose (mmol/L)	5.96 ± 1.58	5.93 ± 1.56	0.158	0.875
Cranial CT			1.945	0.163
Normal (n, %)	2(2.9)	29(7.4)		
Abnormal (n, %)	68(97.1)	364(92.6)		
EEG			3.250	0.071
Normal (n, %)	65 (92.9)	333 (84.7)		
Abnormal (n, %)	5 (7.1)	60 (15.3)		

Abnormal EEG include: Sinus arrhythmia; Ventricular premature beats; Non-sustained ventricular tachycardia; Atrioventricular block

*CT* Computed tomography, *EEG* Electroencephalogram

^*^P-values in bold and italic indicate statistically significant differences

To further understand the correlation between risk factors and FS recurrence, we performed univariate logistic regression. The results showed that age was associated with decreased risk of recurrence within 1 year (OR = 0.61, 95% CI: 0.46, 0.80, *P* < 0.001), the younger the age, the higher the risk of recurrence. The duration of the first episode was associated with increased risk of recurrence (OR = 1.03, 95% CI: 1.00, 1.06, *P* = 0.040). Peak temperature was associated with decreased risk of recurrence (OR = 0.68, 95% CI: 0.47, 0.98, *P* = 0.036). See Table 3 for specific results.

**Table 3:** Univariate Logistic Regression Analysis of Recurrence of Febrile Seizures

Variable	OR	95% CI	*P*
Age (years)	0.61	(0.46, 0.80)	** < *0.001***
Gender	1.17	(0.70, 1.95)	0.549
Duration of the first episode (min)	1.03	(1.00, 1.06)	***0.040***
Peak temperature (℃)	0.68	(0.47, 0.98)	***0.036***
Neutrophil count (10^9/L)	0.94	(0.88, 0.99)	***0.022***
NLR	0.93	(0.86, 1.00)	***0.055***
Serum potassium (mmol/L)	1.63	(1.00, 2.65)	0.0498
Serum sodium (mmol/L)	1.09	(1.01, 1.16)	***0.018***

*NLR* Neutrophil–lymphocyte ratio

^*^P-values in bold and italic indicate statistically significant differences

**Fig. 1:**
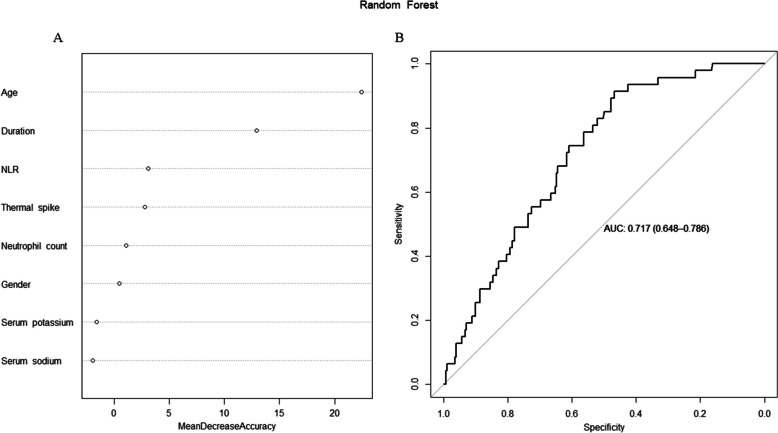
**A** Febrile seizures recurrence random forest weights plot; **B** Febrile seizures recurrence random forest ROC curve. Duration: Duration of the first episode; NLR: Neutrophil–lymphocyte ratio; Thermal spike: peak body temperature before the first episode

A random forest model was constructed by using age, gender, duration of the first episode, peak temperature, neutrophil count, NLR, serum potassium and serum sodium as characteristic values. The results indicated that age was the most significant predictor of FS recurrence, followed by the duration of the first episode. The Random Forest model achieved high accuracy in predicting FS recurrence, as evidenced by an area under the ROC curve of 0.717. The random forest weights and ROC curves are shown in Fig. 1.

## Discussion

FS are a common type of seizures in children and a frequent cause for emergency department visits among children. They typically manifest as seizures accompanied by a rapid increase in body temperature (≥ 38 °C). Diagnosis requires ruling out intracranial infections and other organic or metabolic disorders that may cause seizures. The precise pathogenesis of FS remains inadequately elucidated; however, consensus among experts suggests that it is multifactorial, involving genetics, neurological development, viral infections, and inflammatory responses [2, 7, 22, 23]. Although the majority of children with FS exhibit a favorable prognosis following timely symptomatic intervention, a subset encounter recurrence and manifest a less favorable outcome. Compared with In contrast to approximately 30% recurrence rate reported in studies in Western world, the 1-year recurrence rate observed in the present study stood at 15%, a figure similar to the results of another study involving children from southern China [14]. Age, duration of first episode, peak temperature, neutrophil count, NLR, serum potassium, and serum sodium were found to be possibly associated with FS recurrence in this study.

Our study revealed that children in the recurrent group were significantly younger than those in the non-recurrent group. Fetveit et al. identified 18 months as the peak age of onset for first FS, with a higher likelihood of recurrence in children under 1 year old [23]. Other studies have also established age as an independent risk factor for recurrence after the first episode, with younger age correlating with a higher recurrence probability [5, 6, 14, 24, 25]. The impact of age on FS recurrence may be linked to the development of the nervous and immune systems. Younger children tend to have less developed myelin sheaths and less effective excitatory-inhibitory brain functions, leading to a lower seizure threshold, greater sensitivity to temperature changes, and lower tolerance to fever. This may explain why the peak temperature during the first episode was lower in the recurrent group compared to the non-recurrent group in our study. A large cohort study also reported that the risk of recurrence decreased with higher body temperatures, showing an average 18% reduction in the FS recurrence rate per 1℉ increases. Another prospective study also suggests that a relatively low temperature prior to the initial seizure is a strong predictor of three or more recurrences [25].

The impact of gender on FS recurrence lacks consensus in the literature. Kazemi et al. reported a higher likelihood of recurrence among boys in their study [6], and Agrawal et al. also identified males as a major risk factor for recurrence [18]. In contrast, Berg et al.'s prospective follow-up study of 347 children over a two-month period found no significant difference in recurrence risk between genders [26]. Similarly, Berg et al.'s subsequent cohort study with a larger sample size and longer follow-up (2 years or more) did not observe a association between gender and FS recurrence [19]. Consistent with these findings, present study also found no association between gender and FS recurrence. These differing results may be attributed to variations in study populations and sample sizes.

The duration of the first episode also plays a pivotal role as a risk factor. Our study revealed that patients with a longer duration of the first episode (Mean = 6.1 min) had a higher likelihood of recurrence, consistent with prior research [6]. FS are believed to arise from abnormal brain discharges due to immature brain development and incomplete myelination in children. The seizure process involves muscular convulsions and respiratory abnormalities, and hypoxia resulting from prolonged or sustained convulsive seizures can lead to selective hippocampal neuronal necrosis and apoptosis. This irreversible brain damage may further elevate the risk of FS, establishing a vicious cycle [27].

The association between serum sodium levels and FS remains a contentious issue. Some studies, as well as the AAP, have noted lower serum sodium levels in children with FS compared to controls. Moreover, some research has linked FS to mutations in genes responsible for sodium channel receptors [28]. However, the regular assessment of serum electrolytes is not currently recommended [1, 29].The association between serum potassium and FS remains ambiguous. While a study reported a significant association between serum sodium levels and FS recurrence [30], this finding faced challenge [31]. In our study, serum sodium and potassium exhibited an associated with FS recurrence in univariate logistic regression. However, the result requires further validation. As highlighted by other researchers, the values of serum sodium, potassium, and other electrolyte measurements in children of different ages may not accurately capture their true relationship with FS recurrence, the presence of numerous confounding factors.

The association between FS and inflammatory factors has been increasingly studied. NLR has been recognized as a risk factor for FS in multiple studies, including meta-analyses, and is now regarded as a cost-effective diagnostic biomarker for the disease [16, 17, 32–34]. Several studies have also reported an association between NLR and FS recurrence. Bril et al. found that an elevated NLR posed a significant risk for FS recurrence (OR = 2.64, 95% CI: 1.17–4.85) [17]. In our study, Neutrophil count was significantly different between two groups (*P* = 0.007)with lower level observed in the recurrent group compare to the non-recurrent group. Through univariate logistic regression, we observed a marginal association between NLR and FS recurrence (OR = 0.93, 95% CI: 0.88–0.99, *P* = 0.055), which contradicted previous findings. Although NLR is widely recognized as a cost-effective and accessible diagnostic biomarker marker for FS, most recent studies do not mention the association between NLR and FS recurrence. The results of the random forest model in this study suggest NLR might not hold indispensable significance in predicting FS recurrence. Therefore, additional validation of NLR as a risk factor for predicting FS recurrence is necessary.

In this study, we implemented a random forest model to predict FS recurrence, a machine learning algorithm that uses Bagging (Bootstrap aggregating) combined with decision trees to reduce overfitting and provide comprehensive data prediction. The model included age, gender, duration of the first episode, peak temperature, neutrophil count, NLR, serum potassium and sodiumbased on statistical results and practical clinical applications, with risk factors ranked by their importance scores. Age emerged as the most crucial factor in predicting FS recurrence, followed by duration of the first episode. The relative importance of NLR was significantly lower than the duration of the first episode, while serum sodium and potassium levels were of lesser significance. The results suggest that age and duration of the first episode are the primary considerations for assessing the risk of recurrence in children, consistent with previous research findings. NLR and peak temperature can serve as supplementary indicators, while serum electrolytes and gender do not play a pivotal role in predicting FS recurrence.

This study presents a preliminary analysis of risk factors for the recurrence of FS in children in Nantong, China, with the goal of developing a prediction model and clinical assessment system for FS recurrence. Our sample included 463 cases with a one-year follow-up, a feature not commonly found in many other studies. Our study did not yield positive results for the family history of FS or epilepsy, which is a common focus in most studies. This may be due to the way questions about family history = were framedduring follow-up, resulting in a lack of accurate data. Additionally, as mentioned previously, FS is influenced by various factors, including heredity, genes, and neurological development, which were not fully explored in our study. Future research should involve larger sample sizes, longer follow-up periods, and account for more confounders.

## Conclusion

The recurrence rate of FS within 1 year in children in Nantong, China, is about 15%. Young age and long duration of the first episode are significant independent risk factors for FS recurrence, serving as primary reference factors in predicting recurrence. The potential use of NLR as a predictor of FS recurrence needs further validation.

## Data Availability

The raw data involved in the experiments can be obtained by request to the corresponding author via email.

## Abbreviations

AAP: The American Academy of Pediatrics
IL-6: Interleukin-6
IL-1β: Interleukin-1β
TNFα: Tumor necrosis factor-alpha
NLR: Neutrophil–Lymphocyte Ratios
ROC: Receiver operating characteristic
